# Casein Kinase 1 and Human Disease: Insights From the Circadian Phosphoswitch

**DOI:** 10.3389/fmolb.2022.911764

**Published:** 2022-06-03

**Authors:** Joel C. Francisco, David M. Virshup

**Affiliations:** ^1^ Program in Cancer and Stem Cell Biology, Duke-NUS Medical School, Singapore, Singapore; ^2^ Department of Pediatrics, Duke University School of Medicine, Durham, NC, United States

**Keywords:** casein kinase 1 (CK1), circadian rhythms, sleep disorder (SD), protein phosphorylation / dephosphorylation, cancer, drug addiction, alzheimer disease

## Abstract

Biological systems operate in constant communication through shared components and feedback from changes in the environment. Casein kinase 1 (CK1) is a family of protein kinases that functions in diverse biological pathways and its regulation is beginning to be understood. The several isoforms of CK1 take part in key steps of processes including protein translation, cell-cell interactions, synaptic dopaminergic signaling and circadian rhythms. While CK1 mutations are rarely the primary drivers of disease, the kinases are often found to play an accessory role in metabolic disorders and cancers. In these settings, the dysregulation of CK1 coincides with increased disease severity. Among kinases, CK1 is unique in that its substrate specificity changes dramatically with its own phosphorylation state. Understanding the process that governs CK1 substrate selection is thus useful in identifying its role in various ailments. An illustrative example is the PERIOD2 (PER2) phosphoswitch, where CK1δ/ε kinase activity can be varied between three different substrate motifs to regulate the circadian clock.

## The CK1 Gene Family, With a Focus on CK1δ/ε

**FIGURE 1:**
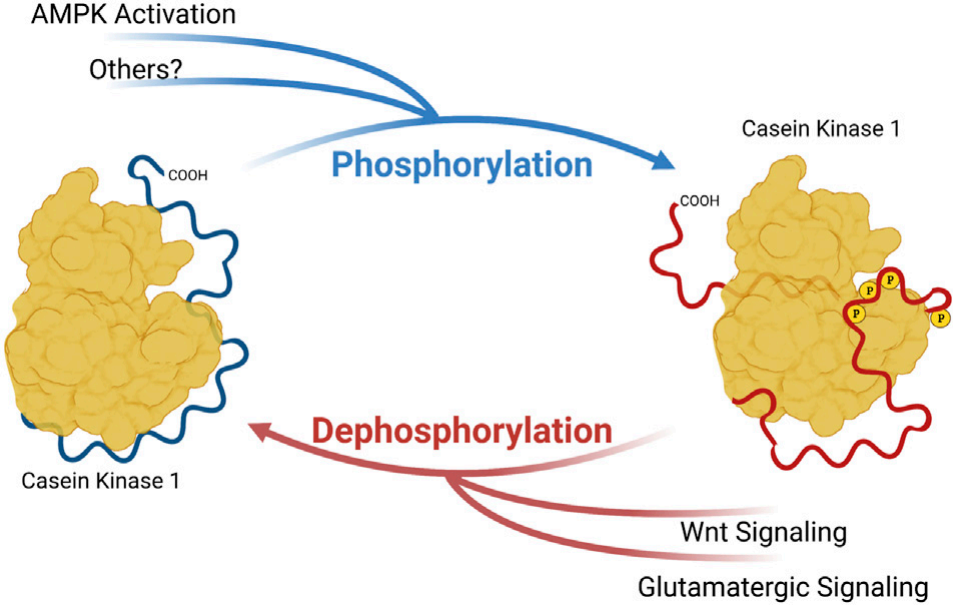
Phosphorylation of Casein Kinase 1 Carboxyterminal tail influences substrate preference. Phosphoryl groups on the carboxyterminal tail may interact with anion-binding pockets on CK1, altering the conformation of substrate binding cleft and substrate preference. Created with Biorender.

CK1δ and CK1ε are members of a family of serine/threonine kinases that are highly conserved among eukaryotes. In humans, this group of kinases is encoded by six distinct genes, CSNK1A/D/E/G1/G2/G3 (Casein Kinase 1 α, δ, ε, γ-1, γ-2, and γ-3). Of these isoforms, CK1δ and CK1ε are the most closely related, sharing over 96% sequence identity in the kinase body, and have very similar carboxyterminal extensions of ∼124 amino acids. CK1δ and CK1ε bind to and phosphorylate many of the same substrates, but with some subtle and important differences. The major regulation of CK1δ/ε comes from their C-terminal tails. These CK1δ/ε C-terminal tails are differentially phosphorylated and dephosphorylated, and it has been long known that this tail phosphorylation regulates kinase activity (Gietzen and Virshup, 1999; Graves and Roach, 1995; Rivers et al., 1998). Importantly, diverse signals, including metabotropic glutamate agonists, Wnts and the AMPK pathway regulate the activity of CK1δ/ε through changes in C-terminal tail phosphorylation (Liu F. et al., 2002; Swiatek et al., 2004; Um et al., 2007) (Figure 1). Most recently, subtle changes in the phosphorylation of CK1δ/ε C-terminal tails has been shown to change CK1 preference for distinct sites within one prominent substrate, PER2 (Fustin et al., 2018; Narasimamurthy et al., 2018).

### CK1 Substrate Sequences

CK1 is best known for its preference to phosphorylate acidic substrates. The first-identified CK1 targets are either preceded by acidic residues, or primed by a phosphorylated serine or threonine. The acidic or phosphorylated amino acid is usually three residues upstream of a CK1 target serine/threonine (Flotow and Roach, 1989). Repeats of this CK1 motif are found in many CK1 substrates in the pattern pSxx (S/T)xx (S/T)xx (S/T) and the phosphorylation of this upstream S/T allows propagation of the signal to multiple downstream serine/threonine residues. This phosphorylation of a primed substrate is very rapid. However, CK1 has also been shown to phosphorylate unprimed substrates. These events, while significantly slower than processive phosphorylation of a primed substrate, clearly have important regulatory functions, serving as rate-limiting steps for downstream phosphorylation. The most prominent examples of this principle of CK1 as rate limiting is the case of S45 of β-catenin (Amit et al., 2002; Liu C. et al., 2002; Marin et al., 2003) and S662 of PERIOD2 (PER2) (Toh et al., 2001). Thus, it is important to understand what leads CK1 to prioritize these different “slow” substrate motifs.

Interest in targeting CK1 for therapeutic intervention of sleep disorders, cancer and drug addiction has grown over the years. Multiple CK1 inhibitors have recently been developed and tested *in vitro* and in animal models. The modelling of how CK1δ/ε selects sites on PER2 could provide a better understanding of how CK1 phosphorylate its other substrates. Here we review selected literature describing the phosphoswitch mechanism of PER2 and highlight several diseases where a similar CK1 substrate selection mechanism might be involved.

### Modelling CK1δ/ε Regulation: Lessons From the Circadian Phosphoswitch

While the 24-h rhythmicity of many physiological processes has been observed long before the dawn of modern biology, its genetic basis was first confirmed in 1971 by Konopka and Benzer (Konopka and Benzer, 1971). Using a forward genetic approach in *D. melanogaster,* they identified mutations in a single gene that altered both the time-of-day when flies would emerge from their pupae and locomotor rhythms in adult flies. This gene was named Period (Per) and the rise and fall of its mRNA and protein abundance across the day results in the periodicity of animal clocks. Price, Kloss then identified that the gene Doubletime (dbt), encoding a CK1 orthologue in *Drosophila*, regulated Per degradation via direct phosphorylation (Kloss et al., 1998; Price et al., 1998). In 1999, Ptacek and others identified the first genetically linked sleep disorder in humans (Jones et al., 1999). It was characterized by an abnormally early onset of sleepiness and pre-dawn wakefulness. and so was termed Familial Advanced Sleep Phase (FASP).

The details of the mutation causing FASP has provided great insights into how CK1 interacts with the PERIOD protein. A serine to glycine substitution at residue 662 of PER2 blocked its phosphorylation by CK1 on a series of downstream serine residues, resulting in a decrease of PER2 stability (Toh et al., 2001). Thus, the multiphosphoserine region of Serine-662, -665, -668, -671, and -673, responsible for PER2 stability came to be known as the FASP domain. Phosphorylation of the FASP domain is a 2-step process where a slow priming phosphorylation of S662 creates an ideal CK1 substrate, enabling the subsequent rapid phosphorylation of the downstream serines (Narasimamurthy et al., 2018). Thus, CK1 has two types of substrate recognition in the FASP domain. First, it slowly phosphorylates S662, and then very rapidly phosphorylates the remainder of the FASP multi-serine domain.

**FIGURE 2:**
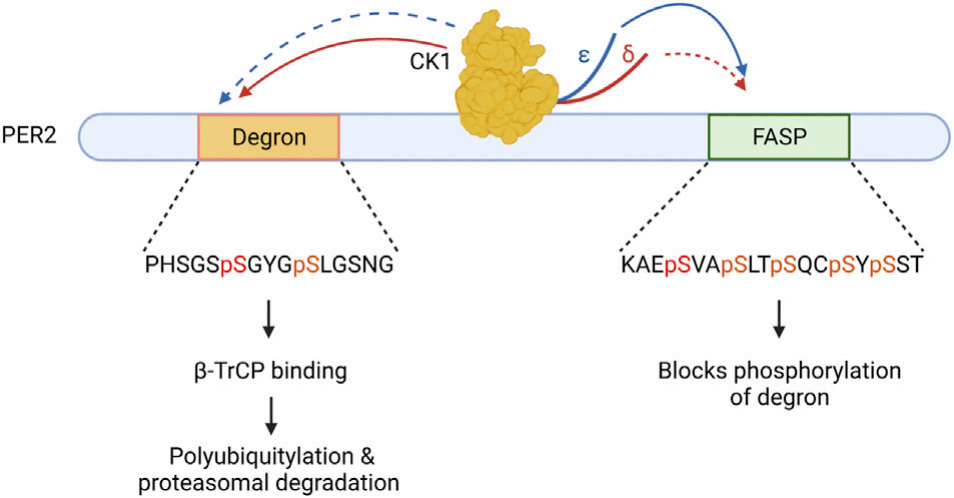
The Circadian Phosphoswitch. CK1 phosphorylation of the PER2 degron results in B-TrCP mediated polyubiquitylation, marking it for degradation by the proteasome. CK1 phosphorylation of the FASP domain stabilizes PER2 by blocking degron phosphorylation. CK1δ (red) and CK1ε (blue) have different preferences for these domains due to non-identical regulatory C-terminal tails. CK1ε displays stronger kinase activity (solid arrow) on the FASP and a weaker kinase activity (dotted arrow) on the degron as compared to CK1δ.

CK1δ/ε have a third type of important substrate recognition in a distant region of PER2, a domain referred to as the phosphodegron. CK1 phosphorylation of the human PER2 phosphodegron at S480 and S484 allows for binding of β-TrCP, an E3 ubiquitin ligase, which then leads to polyubiquitylation and proteasomal degradation of PER2 (Eide et al., 2005; Reischl et al., 2007). Importantly, CK1 phosphorylation of FASP domain of PER2 prevents phosphorylation of the phosphodegron. Thus, CK1 must choose–phosphorylate FASP and stabilize PER2, or phosphorylate the phosphodegron and destabilize PER2. This mechanism, termed the phosphoswitch model of PER2 regulation (Figure 2), allows fine tuning of CK1 substrate specificity within the PER2 protein to increase stability or drive degradation (Zhou et al., 2015; Masuda et al., 2020).

We have seen that within the PER2 phosphoswitch, there are three distinct substrate motifs for CK1: the slow priming site of the FASP, the rapid four phosphoserine residues downstream of the FASP, and the slow phosphodegron. Using x-ray crystallography, Philpott et al. described a two-state activation loop in the CK1δ/ε kinase that contributes to substrate selection (Philpott et al., 2020a). Molecular dynamics simulations of the loop found that it resided predominantly in the “Loop Down” position, but it could enter the “Loop Up” position on rare occasion. Flipping the loop up was predicted to reduce the volume of the CK1 substrate binding cleft allosterically. Importantly, the loop down versus loop up configuration could be controlled by changes in the anion environment, and in the occupancy of anion binding pockets on the kinase body. Given that phosphorylation of the CK1 carboxyterminal tail creates anionic (phospho-) patches, and that phosphorylated peptides can bind to these anion binding pockets (Gebel et al., 2020), we have proposed that phosphoresidues in the CK1δ/ε tail interact with the anion binding pockets to alter activation loop conformation (Narasimamurthy and Virshup, 2021). Supporting this hypothesis, it was demonstrated that a 15 amino acid deletion from the tail of CK1δ more than doubled its kinase activity on FASP peptide to match CK1ε (fustin 2018) (Narasimamurthy et al., 2018). Notably, this deletion removed two phosphoserine residues from the CK1δ tail which the CK1ε isoform lacks. Similarly, a splice variant of CK1δ altering the extreme C-terminus differentially altered activity on PER2 sites (Fustin et al., 2018). Thus, changes from CK1δ to CK1ε, and changes in CK1δ/ε tail phosphorylation is likely to be a mechanism to alter kinase substrate preference and give CK1 its switch-like behavior. While more insight into the control of CK1 tail phosphorylation and its subsequent interaction with the kinase domain is needed to gain a complete understanding of this process, these findings bring us closer to understanding the regulation of CK1 activity.

### CK1 - Period and Sleep Disorders

The characterization of FASP syndrome demonstrates that regulation of Per2 abundance by CK1 is integral in maintaining normal sleep-wake physiology. Consistent with this, mutations in the CK1 gene also cause sleep disorders. In 1988, a mutation called Tau was identified in Syrian golden hamsters that caused a shortening of the circadian period. Heterozygous or homozygous Tau animals experienced a 22-h or 20-h biological day as compared to the standard 24-h of the wildtype controls (Ralph and Menaker, 1988). 12 years later, Lowrey and others traced the cause of this phenotype to a single R178C substitution in CK1ε which abolishes one of the anion binding pockets described above. This mutation resulted in an 8-fold reduction of kinase activity on primed FASP peptide of PER2 (Lowrey et al., 2000) but increased phosphorylation of the phosphodegron (Philpott et al., 2020b). Thus, abnormalities in CK1 function could cause circadian disruption as mutations in mammals, similar to *Drosophila*.

Human mutations in CK1δ and CK1ε also alter circadian rhythms. Individuals with Delayed Sleep Phase and Non-24-Hour Sleep-Wake Disorder were half as likely to possess at least one allele of a S408N polymorphism in CK1ε (Takano et al., 2004). Notably, S408 is located on the C-terminal tail of CK1ε and is a known phosphorylation site. This S408N variant increased the activity of CK1ε on the FASP peptide, consistent with earlier findings that S408 phosphorylation contributed to autoinhibition of CK1ε (Gietzen and Virshup, 1999; Takano et al., 2004). Another family with FASP had aCK1δ T44A mutation that caused reduced kinase activity on PER substrates (Xu et al., 2005). While the phenotype was not observed to be as dramatic as the CK1ε^Tau^ mutant, it was also linked to migraine in the same family (Brennan et al., 2013). These studies confirm that CK1 is a critical core circadian protein whose mutation leads to circadian related disorders. The dysregulation of CK1 activity has also been implicated in other diseases via additional substrates (Table 1).

**TABLE 1:** CK1 target sites in PER2, Connexin-43, 4EBP1 and DARPP32.

Human CK1 target	Residues	Unprimed	Primed	Function
PER2 degron	S-480, 484	PHSGSpSGYGSLGSNG	PHSGSpSGYGpSLGSNG	β-TrCP dependent polyubiquitination and proteasomal degradation
PER2 FASP	S-662, 665, 668, 671, 674	PGKAEpSVASLTSQCSYSSTIVH	PGKAEpSVApSLTpSQCpSYSpSTIVH	Protein stabilization by blocking degron phosphorylation
CX-43	S-325, 328, 330	MGQAGpSTISNSHAQPF	MGQAGpSTIpSNpSHAQPF	GJ assembly and membrane localization
4EBP1	T-41, 50	TTPGGpTLFSTTPGGpTRIIYD		Release of EIF4e for cap-dependent translation
DARPP-32	S-137	PPLDEpSERDGG		Enhance PP1 inhibitory effect; facilitate switching to PKA inhibition

Amit, S., hatzubai, A., birman, Y., andersen, J.S., Ben-Shushan, E., mann, M., Ben-Neriah, Y., and Alkalay, I (2002). Axin-mediated CKI, phosphorylation of β-catenin at Ser 45: a molecular switch for the Wnt pathway. Gene Dev *16*, 1,066–1,076.

### CK1 Regulates Cell-Cell Interactions Through Connexin-43

Hyperphosphorylation of Connexin-43 (Cx-43) by CK1 may contribute to tumor metastasis in pancreatic and breast cancers. Under normal conditions, CK1 helps maintain cell-cell interfaces in part by phosphorylating Cx-43, a key structural protein of gap junctions (GJ). These structures facilitate cell-cell communication by allowing the exchange of small molecules and ions through their pore-like hemichannels. GJs are also known to transduce signals from the cell membrane to alter transcriptional programs. This includes signals that promote extravasation, cell migration, differentiation, and apoptosis. Therefore, Cx-43 can both prevent and promote tumorigenesis depending on its regulation.

Cooper et al., identified a multi-phosphoserine domain in Cx-43 that was phosphorylated by CK1δ/ε (Cooper and Lampe, 2002). This phosphodomain is important for the membrane localization of Cx-43 and assembly of GJ complexes, and inhibition of CK1δ/ε activity interfered with GJ formation (Cooper and Lampe, 2002). In epithelial cells, mutation of the Cx-43CK1 target serines to alanines, creating Cx-43^CK1A^, reduced cell migration in wound healing assays. Correspondingly, serine to glutamic acid substitutions to generate phosphomimetic Cx-43 at these sites caused increased migration (Lastwika et al., 2019). This property of Cx-43 was unique to the CK1-regulated phosphodomain. Hence, dysregulation of this interaction could disrupt cell signaling and migration, both of which play important roles in cancer.

### Ck1, Cx-43 and Cancer

Recent work has shown that this CK1-CX-43 axis contributes to the severity of pancreatic ductal adenocarcinomas (PDAC), a form of cancer known for its aggressive metastatic ability. In 2021, Solan et al., showed that in a KRAS^G12D^ mouse model of PDAC, phosphorylation of Cx-43 by CK1 was important for epithelial-mesenchymal transition (EMT) (Solan et al., 2021). KRAS^G12D^ mice with the Cx-43^CK1A^ mutations had reduced metastatic burden and increased overall survival compared to KRAS^G12D^ alone. They also observed that primary tumors in KRAS^G12D^; Cx-43^CK1A^ mice had less gap junctions, developed a cyst-like phenotype and maintained expression of epithelial markers, consistent with reduced EMT and less migration.

CK1-enhanced GJ activity may be a feature of other highly metastatic tumors. Rosenberg et al. found that suppressing CK1δ activity with the potent CK1δ/ε inhibitor Sr-3029 reduced the tumorigenicity of Triple Negative Breast Cancer (TNBC) cells (Rosenberg et al., 2015). In the TNBC line MDA-MB231, both CK1δ-specific shRNA and Sr-3029 treatment led to a reduction in cell proliferation. This result was not observed in CK1δ-low breast cancer lines like MCF7. In mouse orthotopic xenografts, Sr-3029 treatment drastically reduced the volume of MDA-MB231 tumors. Subsequent work by Bar et al. confirmed these findings (Bar et al., 2018). They similarly found that CK1δ knockdown MDA-MB231 resulted in significantly slower growing tumors. Additionally, they showed that CK1δ promoted MDA-MB231 cell migration. CK1δ knockdown tumors displayed decreased ability to metastasize both *in vivo* and *ex vivo*. Although CX-43 phosphorylation was not directly assessed in this model, they noted repression of junctional proteins in these breast cancer models, making CX-43 an interesting candidate for further study.

### CK1 Regulates Protein Translation via 4EBP1

There is growing interest in the use of translation inhibition as a therapy for cancer. Due to its critical role in cell growth, translation is a tightly regulated process. Most of this control takes place at the translation initiation phase and is regulated by the heterotrimeric Elongation Initiation Factor 4a Complex (EIF4A). Elongation Initiation Factor 4e (EIF4E) is a rate-limiting component of the EIF4A complex. It functions to bind the m7g cap of mRNA to recruit it to the ribosome for translation. Suppressing translation is thus accomplished through the sequestration of EIF4E by 4 E-binding protein 1 (4EBP1). 4EBP1 phosphorylation releases EIF4E to drive translation, so the kinases that phosphorylate 4EBP1 are of great interest. While initial studies on focused on the mTOR and PIK3-regulated phosphorylation, more recent studies have found that CK1δ/ε also phosphorylates 4EBP1 on a distinct set of regulatory sites, also leading to EIF4E release (Brunn et al., 1997; Gingras et al., 1999, 2001).. (Shin et al., 2014; Deng et al., 2017)

How might the activity of CK1 on 4EBP1 be regulated? One possibility is via AMP-activated protein kinase (AMPK). AMPK is a cellular energy sensor that shuts down protein translation when intracellular ATP stores are depleted. AMPK has been shown to activate CK1ε by phosphorylating its regulatory tail on S389 (Um et al., 2007). Based on what we now know about CK1δ/ε regulation, this suggests that phosphorylation of this site on CK1 may bias the substrate selection of the kinase. (Fustin et al., 2018; Narasimamurthy et al., 2018). We speculate that cells could also use AMPK phosphorylation of CK1ε to change its substrate selection on 4EBP1.

### CK1, 4EBP1 and Cancer

While CK1 is rarely the primary driver of disease, elevated CK1δ/ε has been associated with highly malignant cancers (Toyoshima et al., 2012; Rosenberg et al., 2015; Bar et al., 2018). One potential target of this increased CK1 activity is the translation of c-Myc oncogene. Recent work by Deng et al. successfully utilized inhibition of CK1ε to treat Myc-translocation-positive lymphomas via the suppression of protein translation (Deng et al., 2017). The *MYC* proto-oncogene encodes a transcription factor that influences the expression of around 15% of total human transcripts. Under normal circumstances, *MYC* is under tight transcriptional and translational regulation, and MYC protein is quickly degraded to prevent its accumulation. However, MYC overexpression is detected in a majority of cancers and correlates with poor treatment outcomes. *MYC* gene amplification and translocation cancers are particularly aggressive and challenging to treat, owing to the undruggable nature of Myc protein (Mossafa et al., 2009; Jonge et al., 2016). As part of its regulation, the 5’ UTR of Myc mRNA forms complex secondary structures, making it heavily dependent on the EIF4A complex for translation (Wolfe et al., 2014). Thus, Deng and others surmised that stopping EIF4E release from 4EBP1 might be sufficient to block hypertranslation of Myc. To do this, they developed a PI3Kδ (IC_50_ = 22 nM) and CK1ε (IC_50_ = 6 uM) dual kinase inhibitor, umbralisib (TGR-1202). Umbralisib gave promising results in reducing viability of multiple MYC-driven lymphomas. It was found to out-perform idelalisib, the best-in-class approved PI3Kδ inhibitor. Umbralisib treatment greatly reduced C-MYC protein levels by effectively blocking the release of EIF4E from 4EBP1. This effect was reproduced when the lymphoma cells were treated with idelalisib in combination with the CK1ε specific inhibitor PF4800567, showing that co-inhibition of PI3KD and CK1ε was required to achieve potency. Interestingly, Maharaj et al., showed that the CK1ε inhibitory ability of umbralisib contributes to the safety profile of the drug (Maharaj et al., 2020). This was observed when either umbralisib alone or idelalisib with PF480567 preserved a healthy population of regulatory T-cells (Tregs) in mice while treatment idelalisib alone did not. The authors proposed that dual-inhibition of PI3Kδ and CK1ε reduces immune mediated toxicities resulting in lower risks of treatment. Thus far, this method of translation inhibition has proven to be well tolerated among patients. The FDA has recently granted accelerated approval for the drug umbralisib in the treatment of Marginal Zone Lymphoma and Follicular Lymphoma while several phase 2 and 3 trials are underway for other forms of lymphoma (https://clinicaltrials.gov).

Bryja and coworkers recently demonstrated the potential of CK1 inhibition in Chronic Lymphocytic Leukemia (CLL) (Janovska et al., 2018). Treatment in a mouse model of CLL, Eμ-TCL1, with PF670462, a dual CK1δ/ε inhibitor, was found to significantly delay disease onset and extend overall survival. While the authors note that CK1 inhibition is likely non-curative in leukemia, it has strong therapeutic potential in combination with other tumor suppressive drugs. Although the role of 4EBP1 in CLL is unclear, this is a fruitful area for study given the safety and effectiveness of umbralisib in a closely related hematological malignancy.

### Ck1, 4EBP1, and Metabolic Disorders

Could CK1δ/ε regulate translation of other important genes? A growing body of research has identified CK1 as a contributing factor to various metabolic disorders. One example is its role in promoting the maturation of pre-adipocytes in white adipose tissue (WAT) via EIF4E-dependent translation. This proliferation of WAT heightens the risk of disease by enhancing hunger signals, inducing insulin resistance, raising basal inflammation and altering sleep behavior. While PPARγ and C/EBPs transcription factors are considered the master regulators of adipocyte differentiation, they are in turn regulated by the cellular energy sensor, AMPK (Sozio et al., 2011; Cheang et al., 2014; Mancini et al., 2017). As with *MYC*, PPARγ translation is also tightly regulated by highly stable secondary structures in its 5’ UTR (McClelland et al., 2009). Thus, adipogenesis is dependent on EIF4A complex activity and hence sensitive to EIF4E sequestration by 4EBP1 (Tsukiyama-Kohara et al., 2013; Tsai et al., 2016). CK1 inhibitors are therefore potential candidates for obesity intervention. Indeed, CK1δ/ε inhibition with the small molecule inhibitor, PF5006739, was shown to improve glucose tolerance in both diet-induced and genetic models of obesity in mice (Wager et al., 2014; Cunningham et al., 2016), an effect that we speculate is due in part to decreased translation of PPARγ.

Yang et al. presented additional circumstantial evidence supporting the idea that CK1δ/ε activity regulates PPARγ translation (Yang et al., 2019). They found the soy derived compound, Orobol, reduced accumulation of WAT in mice fed with a high fat diet. It also prevented phosphorylation of 4EBP1 at serine-65 and reduced expression of PPARγ in pre-adipocytes. Kinome screening identified CK1ε (IC_50_ = 1.24 uM) as the kinase most sensitive to Orobol treatment. However, Orobol also inhibited MAP4K5 (IC_50_ = 1.48 uM), which also has the potential to affect 4EBP1 phosphorylation via the mTOR pathway (Gingras et al., 1999).

### CK1 and DARPP-32

DARPP-32 was discovered due to its role in dopaminergic signaling, a key regulator of neuronal development, memory and learning that acts through the transcription factor CREB. This process is characterized by the stimulation of dopamine D1 receptors, activating Protein Kinase A (PKA), which in turn activates CREB through phosphorylation. Dephosphorylation of CREB by Protein Phosphatase 1 (PP1) attenuates this pathway. Another target of PKA is Dopamine- and cAMP-Regulated Neuronal Phosphoprotein of 32kDa, DARPP-32. PKA phosphorylates DARPP-32 on Threonine-34, turning it into a potent inhibitor of Protein Phosphatase 1 (PP1), thus strengthening CREB-dependent transcription. Activation of metabotropic glutamate receptors (mGluR), which are known to enhance dopaminergic signaling, decreases phosphorylation of CK1 C-terminal tail by activation of calcineurin. This increases CK1 activity on S137 of DARPP-32, which protects dephosphorylation of T-34 from Calcineurin, further strengthening suppression of PP1(Desdouits et al., 1995; Liu F. et al., 2002). DARPP-32 is also known to be phosphorylated by CDK5 on Threonine-75 which switches it into an inhibitor of PKA. Interestingly, CK1 phosphorylation of S137 is a pre-requisite for CDK5 phosphorylation of DARPP-32. This implies that CK1 plays a critical role in DARPP-32’s conversion from PP1 to PKA inhibitor. Of note, S137 is situated in a highly acidic region of DARPP-32. Thus, phosphorylation of CK1 could limit its accessibility to the DARPP-32 substrate site, amplifying dopamine D1 receptor signaling and increasing drug sensitivity.

### CK1, DARPP-32 and Drug Addiction

Neuroadaptations to the dopamine signaling system from substance abuse leads to drug dependence (Thomas et al., 2008). Since CK1 phosphorylation of DARPP-32 modulates dopaminergic signaling, various studies have explored its role in substance abuse. In mice, DARPP-32 phosphorylation was shown to influence addiction to the habit-forming drug, methamphetamine (MA) (Bryant et al., 2009a; 2012a). Linkage analysis performed in mice bred for high sensitivity to MA-induced locomotor activity revealed Quantitative Trait Loci (QTL) linked to CK1ε overexpression. In these mice, CK1ε transcript was increased by over 10-fold in the nucleus accumbens (Palmer et al., 2005). This suggests a positive relationship between CK1ε activity and MA sensitivity. This was tested by the inhibition of CK1 with PF670462 (CK1δ IC_50_ = 14 nM, CK1ε IC_50_ = 7.7 nM), a dual CK1δ/ε inhibitor, and this blocked MA-induced locomotor activity leading to the conclusion that CK1 promoted drug sensitivity (Bryant et al., 2009b). However, the story got more complicated. In a follow-up study, CK1ε knockout mice displayed increased sensitization to MA (Bryant et al., 2012b). This time, CK1ε-specific inhibition with PF4800567 (IC_50_ = 32 nM) enhanced MA-induced locomotor activity in the MA sensitive mice, suggesting that CK1ε acts to attenuate drug addiction. The differences in substrate preference between CK1δ and CK1ε may account for this contradictory finding. Supporting this theory, Wager et al. observed a suppressive effect on drug-seeking behavior in rats treated with PF50067399 (CK1δ IC_50_ = 3.9 nM, CK1ε IC_50_ = 17 nM), a brain-penetrant dual CK1δ/ε inhibitor (Wager et al., 2014). This molecule is four times more potent on CK1δ than CK1ε. Thus, it appears that while CK1δ enhances drug sensitivity, CK1ε acts to suppress it. Zhou et al. provided additional evidence, showing that mice with forebrain overexpression of CK1δ were more sensitive to amphetamine and methylphenidate, which both stimulate the dopaminergic pathway (Zhou et al., 2010). This suggests that CNS-active CK1δ inhibitors might be useful in the treatment of drug addiction.

### Alzheimer Disease and CK1

Alzheimer Disease (AD) is a neurodegenerative disease characterized by a progressive decline in cognitive functions such as memory, speech, and mood regulation. While the cause of AD remains unknown, disease severity is correlated with the accumulation of hyperphosphorylated Tau protein and amyloid-ß plaques (Aß) which impairs proper neuronal function (Ballard et al., 2011). The failure to produce significant clinical outcomes in patients of AD after Aß plaque clearance highlights the importance of understanding both Tau and Aß accumulation (Sevigny et al., 2016; Selkoe, 2019). One mechanism of Tau hyperphosphorylation is the reduced activity of PP1 in AD brain tissue (Gong et al., 1993; Wang et al., 1996; Sun et al., 2003). Various studies have established that CK1δ and CK1ε protein are both highly upregulated in human AD brain tissue (Ghoshal et al., 1999; Yasojima et al., 2000). Potentially, the increase in CK1δ/ε results in an accumulation of T-34 phosphorylated DARPP-32 and a pathological inhibition of PP1. This is in line with the findings of Sundaram et al., where inhibition of CK1δ/ε with PF670462 improved cognitive function and reduced Aß plaques in a mouse model of AD (Sundaram et al., 2019). Establishing a direct connection between CK1-mediated DARPP-32 phosphorylation and hyperphosphorylated Tau accumulation could provide new insights into developing AD therapies.

### Conclusion and Future Directions

Altered regulation of CK1δ/ε activity is associated with many diseases including sleep disorders, metabolic syndrome, Alzheimer Disease and cancer (Depner et al., 2014; Summa and Turek, 2014; Musiek et al., 2015; Stephenson et al., 2021). However, the molecular pathways that directly control the activity of CK1 in these diseases remain poorly understood. Insights from the regulation of circadian rhythms may expand our understanding of the role of CK1 in other processes.

Casein Kinase 1 is a key regulator of the circadian clock, determining the duration of each biological day through the phosphorylation of at least three unique PER2 domains. The ability of CK1 to choose between these three potential phosphorylation sites is a combination of several regulatory mechanisms. Firstly, the various CK1 isoforms have differing propensities for carboxyterminal tail modification. Secondly, CK1 preference for the substrates are influenced by modifications to its carboxyterminal tail. Finally, the ratio of expression between the various CK1 isoforms (and the CK1δ splice variants) also plays a role. These mechanisms may extend to other disease-relevant substrates including Cx-43, 4EBP1 and DARPP-32. Unlocking the ability to switch CK1 activity between substrates potentially enables the precise modulation of these CK1 target proteins, offering new avenues of therapy for their respective diseases.
